# Patient and family-centered care practices during inter-facility transfer of critically ill children: an exploratory mixed methods study

**DOI:** 10.3389/frhs.2026.1835422

**Published:** 2026-08-20

**Authors:** Claire Stucky, Aaron M. Scherer, Irene Pantekidis, Caroline Elser, Aditya Badheka, Shilpa C. Balikai, Madhuradhar Chegondi, Mitchell A. Luangrath, Nehal R. Parikh, Priyadarshini R. Pennathur, Heather Schacht Reisinger, Christina L. Cifra

**Affiliations:** 1 Des Moines University College of Osteopathic Medicine, West Des Moines, IA, United States; 2 Department of Internal Medicine, University of Iowa Carver College of Medicine, Iowa City, IA, United States; 3 Division of Medical Critical Care, Department of Pediatrics, Boston Children’s Hospital, Boston, MA, United States; 4 Department of Teaching, College of Education, University of Northern Iowa, Cedar Falls, IA, United States; 5 Division of Critical Care Medicine, Department of Pediatrics, University of Iowa Carver College of Medicine, Iowa City, IA, United States; 6 Division of Pediatric Critical Care Medicine, University of Illinois College of Medicine at Peoria and OSF HealthCare Children’s Hospital of Illinois, Peoria, IL, United States; 7 Division of Critical Care Medicine, Department of Pediatrics, Children’s Mercy Hospital Kansas City, University of Missouri-Kansas City School of Medicine, Kansas City, MO, United States; 8 Department of Industrial, Manufacturing, and Systems Engineering, University of Texas at El Paso, El Paso, TX, United States; 9 Department of Internal Medicine, University of Iowa Carver College of Medicine, Implementation Science Center and Institute for Clinical and Translational Science, University of Iowa, Iowa City, IA, United States; 10 Division of Medical Critical Care, Department of Pediatrics, Boston Children’s Hospital, Harvard Medical School, Boston, MA, United States

**Keywords:** critical care, family needs, family-centered care, inter-facility transfers, patient experience

## Abstract

**Introduction:**

The inter-facility transfer of a critically ill child to the pediatric intensive care unit (PICU) is a highly stressful time for families. This exploratory study aimed to identify care practices important to patients/families during urgent transfers of children from frontline settings to a higher level of care.

**Methods:**

We conducted a mixed methods study using surveys and interviews of parents/guardians of children transferred to a single PICU from external institutions. Participants were surveyed about their perceived importance of transfer-related care practices, specifically those addressing families' understanding of transfer, communication, family presence, and patient/family needs. A subset of parents were interviewed to characterize their transfer experience and identify improvement opportunities.

**Results:**

Of 23 patient-parent dyads who consented, 13 parents completed the initial survey, 11 completed the follow-up survey, and four participated in interviews. From the surveys, three care practices were identified as most important to responding families: 1) providing parents/guardians with verbal information about their child's condition, 2) providing patients the ability to request their parents/guardians during transfer, and 3) immediately contacting families with significant patient status changes. Interviews revealed that communication with healthcare teams is a substantial factor affecting families' experience of a smooth vs. stressful inter-facility transfer.

**Conclusions:**

In this exploratory study, care practices ensuring that timely information about a child's clinical condition is relayed to their parents/guardians were most important to families of critically ill children undergoing inter-facility transfer to the PICU. Communication between families and healthcare teams plays a significant role in families' experiences of transfer.

## 1 Introduction

Inter-facility transfers have become more common with the regionalization of subspecialty pediatric care. From 2000 to 2014, the likelihood that a child will receive definitive care at a hospital without need for transfer decreased by 65% (1). Indeed, over 60,000 children are transferred each year from U.S. emergency departments (EDs) to a higher level of care (2). The urgent transfer of a critically ill child to a tertiary referral center is a highly stressful and anxious time for families (3). Transfer decisions usually need to be made quickly, parents may travel to the pediatric intensive care unit (PICU) separate from their child, childcare for siblings must be abruptly arranged, and families often need to work with multiple medical teams and navigate unfamiliar hospital environments, all while contemplating the potential consequences of their child's illness (3, 4). Despite the substantial impact of these transfers on patients and families, little attention has been paid to practices that families find important during the inter-facility transfer process.

Patient-centered care is defined as care that is respectful of and responsive to individual patients' and families' preferences, needs, and values (5). Both professional critical care and emergency medicine organizations have endorsed this approach for many years, since patient and family involvement can profoundly influence clinical decisions and patient outcomes (6–9). Although many EDs and PICUs have incorporated principles of family-centered care into their daily practice, it is unclear what patients and families value specifically during urgent inter-facility transitions, and whether the care they receive aligns with what they value. Our prior work involving interviews with parents revealed that communication failures caused the most distress to families around the time of transfer (10). Research involving families of children with special healthcare needs have shown that shared decision-making, responsiveness to resource needs, and consistent communication are necessary to address during inter-hospital transfers (4). However, there is limited information on patient and family-centered practices for urgent transfers of critically ill children. It is necessary to identify these practices to ensure that care provided during these crucial transitions are responsive to patients' and families' needs. The objective of this exploratory study was to identify care practices that are important to patients and families during urgent transfers of critically ill children from frontline healthcare settings to a higher level of care.

## 2 Methods

We conducted an explanatory sequential mixed methods study using surveys and semi-structured interviews of parents/guardians of children transferred to a single PICU from external referring institutions. This study was reviewed and approved by the University of Iowa Institutional Review Board (IRB ID #202201221). We reported our findings in accordance with the Strengthening the Reporting of Observational Studies (STROBE) (11) and Consolidated Criteria for Reporting Qualitative Research (COREQ) guidelines (12).

### 2.1 Study setting and participants

We conducted the study at an academic tertiary referral mixed medical-surgical and cardiac PICU serving a mostly rural population with a large catchment area. At the time of the study, the PICU had 28 beds and admitted approximately 1,400 children per year, of which 30% were directly transferred to the PICU from more than 50 unique referring institutions. Patients are referred for urgent transfer by clinicians mostly from EDs in referring community hospitals. After the decision to transfer has been made, a verbal handoff then occurs by phone between the referring clinician and receiving PICU physician, wherein the PICU physician agrees with the appropriateness of PICU transfer and makes recommendations for urgent diagnostic testing and management prior to PICU admission (13). Patients transferred to the PICU typically require advanced life support for single or multi-organ failure that cannot be provided in a general ward or community/regional facility. All transfers reflect the clinical judgment of both the referring clinician and receiving PICU physician that the child's condition cannot be fully managed at the referring institution and transfer to the PICU is necessary for advanced care. Majority (67%) of patients are transported by the PICU institution-affiliated critical care transport team, while the rest are transported by a variety of local medical transport companies contracted by referring institutions (14). Parents/guardians mostly accompany patients during transport; otherwise, they travel to the PICU separately via private transportation. Upon arrival to the unit, a brief bedside handoff occurs between the transport and PICU teams. The PICU team then evaluates the patient, develops and carries out initial care plans, and speaks to the parents/guardians.

We included patients 0–17 years old and their parents/guardians who were urgently transferred from external referring institutions to the PICU for an acute medical problem requiring a higher level of care. We excluded patients who were admitted electively (i.e., planned admissions with no acute illness) and those who were not directly transferred from an external institution. Patients were screened and recruited from May 16, 2022 to August 24, 2022. Screening was performed daily wherein new admissions' medical records were reviewed for eligibility per the criteria above. We implemented a purposive sampling approach using a sampling grid to recruit participants with diverse racial/ethnic backgrounds, socio-economic circumstances (based on median household income of home zip code), medical needs (i.e., patients with vs. without complex chronic conditions), and geographic distances to the receiving PICU. As patients were included in the study, their characteristics relevant to purposive sampling were documented in the sampling grid. The grid was then used daily to guide which patients to prioritize for recruitment to ensure broad representation across all categories.

### 2.2 Data collection

From chart reviews, we collected patient demographic and clinical data including age, gender, race/ethnicity, presence of complex chronic conditions, number of past PICU admissions over the last 2 years, median family income in patient's home zip code area, and geographic distance of the patient's home address to the PICU. From surveys (see below), we also collected parent/guardian information including relationship to the patient, highest educational attainment, and employment status.

#### 2.2.1 Surveys

We developed an initial set of patient and family-centered care practices relevant to pediatric inter-facility transfer using various sources, including published original research (4, 15–18), professional society guidelines (8), guidance from patient advocacy-provider group alliances (19, 20), data from our own interviews of parents of children at the time of PICU admission (10), and our own clinical experience as practicing PICU clinicians. We organized all relevant care practices into five domains: 1) family understanding of the need for transfer, 2) communication with patients and families, 3) family presence, 4) patient needs, and 5) family needs (see a detailed description of each care practice in Supplementary Table 1). The care practices were then incorporated into a survey instrument wherein respondents were asked to evaluate each practice relevant to other named practices on a 4-point Likert scale (1-not important, 2-somewhat important, 3-important, 4-very important). For each domain, respondents were also asked to rank which care practice was most and least important to them overall. Finally, respondents were given the opportunity to list additional care practices that were not included in each domain and add other text comments. The survey instrument was reviewed and piloted by a patient partner (parent of a patient with a history of frequent transfers to the PICU) for readability and clarity, and was iteratively revised accordingly. Formal psychometric testing and validation of the survey were not performed.

Surveys were administered to parents/guardians of included patients. Surveys were delivered both electronically and via mailed paper form per participants' preferences. No participant incentives were provided for survey completion. All participants were invited to complete two rounds of surveys. Participants were invited to complete the second survey regardless of whether they completed the first. The first survey aimed to identify which care practices were considered most vs. least important by respondents in each domain relative to other care practices in that domain (Supplementary Figure 1). The second survey aimed to distill care practices which were considered by families as most vs. least important across all domains (Supplementary Figure 2). In the second survey, we only included care practices from the first survey if they were rated by more than 75% of respondents as “important” or “very important” and not rated as the least important practice in that domain by any respondent. The 75% threshold is commonly used in consensus studies such as Delphi studies (21), and is a pragmatic cutoff (not empirically validated). Care practices which generated opposite ratings (i.e., an even or almost even number of respondents indicated that they were important vs. not important) were noted for further investigation in interviews.

#### 2.2.2 Interviews

We developed an interview guide based on survey responses, with the goal of gaining qualitative insight into families' inter-facility transfer experiences and identifying opportunities for improvement in the transfer process. Survey results were integrated into the interview guide to provide parents/guardians the opportunity to further elaborate on the importance of certain practices for their child and family. Interview questions were also developed for parents/guardians to more fully characterize their transfer experience, identify factors that contribute to both positive and negative experiences of transfer, and elicit reasons for why certain care practices are regarded as more or less important (Supplementary Figure 3). All parents/guardians were invited to participate in interviews. Using the guide, a researcher (C.S.) with a background in medical anthropology, trained in qualitative interviewing techniques/analysis, and with no prior involvement in patient care conducted semi-structured interviews by phone and via a secure institutional version of the Zoom™ video-conferencing platform. All participants spoke English; thus, no interpreters were used. All interviews were audio-recorded and transcribed.

### 2.3 Data analysis

Quantitative data were summarized using descriptive statistics, calculating proportions for categorical variables and means and standard deviations (normal distribution) or medians and interquartile ranges (non-normal distribution) for continuous variables. Missing data (<10%) were not included in the analysis; no imputation methods were used. We used Stata 14.2 (StataCorp, 2015, College Station, TX) for quantitative analysis. Qualitative data included free text responses from surveys and information from interviews. We first developed deductive codes (initial codebook) based on the interview guide and then iteratively revised the codebook by adding inductive codes as they were identified. Two researchers (C.S., who conducted the surveys and interviews, and I.P., who had no contact with participants) then coded the data using the constant comparative method, assigning predetermined codes to text segments, reviewing each other's codes, and conducting consensus discussions as needed to achieve agreement. Consensus meetings were also conducted periodically including the two coders and a third research team member (C.L.C.) to review codes and practice reflexivity. Finally, directed content analysis (22) was performed to identify overarching themes across the fully-coded data. Member checking was not performed due to challenges with contacting participants. We used MAXQDA© (VERBI Software, 2022, Berlin, Germany) for qualitative analysis.

## 3 Results

### 3.1 Patient and family characteristics

Of 26 patient-parent dyads originally consented for the study, three withdrew before data collection. Thirteen of 23 (57%) parents responded to the surveys in total. Among these 13 responding dyads, patients had a median age of 4 (IQR 0.7, 10) years with 31% being female. Majority (69%) were White and 15% were Hispanic. More than half (62%) had a complex chronic condition and 23% had at least one PICU admission in the previous two years. Parents who responded to the survey were mostly patients' mothers (77%), had completed a college education (62%), and were employed full-time (69%). Most families (62%) lived in a zip code area with a median household yearly income of $50,000 to $74,999 (36th to 52nd U.S. income percentile) (23). The median geographic distance between families' homes and the PICU was 71 (IQR 54, 88) miles with a median ground travel driving time of 80 (IQR 60, 90) minutes (Table 1). Additional analysis revealed that compared to the group which responded to the surveys, among those who did not respond, there were higher proportions of young and Black children (patients), parents/guardians with lower educational attainment and more unemployment, and families with a lower median household yearly income (Supplementary Table 2).

**Table 1:** Patient and family characteristics.

Characteristics	*n* = 13
Patient characteristics	
Age, years, median (IQR)	4 (0.7, 10)
Female gender, *n* (%)	4 (31)
Race, *n* (%)	
White	9 (69)
Black	0
Asian	1 (8)
More than one race	3 (23)
Ethnicity is Hispanic or Latino, *n* (%)	2 (15)
Has a complex chronic condition, *n* (%)	8 (62)
Had at least one PICU admission in previous 2 years, *n* (%)	3 (23)
Parent/guardian characteristics	
Female gender, *n* (%)	10 (77)
Relationship to patient, *n* (%)	
Mother	10 (77)
Father	3 (23)
Highest educational attainment, *n* (%)	
High school	3 (23)
College	8 (62)
Post-graduate	2 (15)
Employment status, *n* (%)	
Part-time	3 (23)
Full time	9 (69)
Unemployed	1 (8)
Family characteristics	
Median household income by zip code, *n* (%)	
$35,000 to $49,999	1 (8)
$50,000 to $74,999	8 (62)
$75,000 to $99,999	4 (31)
Geographic distance from PICU, miles, median (IQR)	71 (54, 88)
Ground travel time from patient's home to PICU, mins, median (IQR)	80 (60, 90)

IQR, interquartile range; PICU, pediatric intensive care unit.

### 3.2 Importance of patient and family-centered care practices

Thirteen parents responded to the first round of surveys and nine responded to the second round. All respondents to the second round also responded to the first survey. In the first survey, majority of parents indicated the following care practices to be *most important* in each domain: 1) ensuring that the family has a good understanding of the emergent nature of transfer, 2) immediately contacting the family if the patient has a significant status change, 3) facilitating family presence on PICU arrival, 4) ensuring patient comfort throughout the transfer process, 5) providing the family with travel directions and PICU wayfinding, and 6) providing the family with sleeping accommodations on PICU arrival.

In contrast, the following care practices were assessed by parents to be *least important* in each domain: 1) clinicians considering the impact of transfer on the family, 2) providing the family with written information and/or updates during transport, 3) facilitating family presence during transport, 4) allowing the patient to bring comfort items during transfer, and 5) providing the family with spiritual resources.

Notably, there were some care practices wherein respondents were *divided as to their importance*. These included: 1) providing an opportunity for the family to meet the transport crew, 2) providing interpreters if needed, 3) facilitating family presence during resuscitation, 4) providing social work assistance soon after PICU arrival, and 5) providing housing resources (Table 2).

**Table 2:** Importance of patient and family-centered care practices from first survey round.

Domain	Care practices	Mean ranking of importance^a^ mean (SD)	Ranked most important *n* (%)	Ranked least important *n* (%)
		*n* = 13		
Family/patient understanding regarding the patient's condition, reason for transfer, and the transfer process	Ensuring that the family understands the:			
	Reason for transfer	3.82 (0.40)	2 (20)	0
	Emergent nature of transfer	3.73 (0.47)	5 (50)	0
	Risk-benefit of transport	3.73 (0.47)	0	1 (10)
	Process of transfer	3.27 (1.00)	1 (10)	3 (30)
	Providing an opportunity for family to meet the transport crew	3.73 (0.47)	2 (20)	2 (20)
	Considering the impact of transfer on family	3.18 (1.08)	0	4 (40)
Communication with family/patient	Immediately contacting the family with status change	4.00 (0)	7 (58)	0
	Providing the family with:			
	Information on medical procedures	3.92 (0.28)	2 (17)	0
	Verbal information on their child's condition	3.77 (0.44)	0	0
	PICU contact information	3.54 (0.78)	1 (8)	1 (9)
	Updates during transport	3.46 (0.97)	1 (8)	4 (37)
	Interpreters if needed	3.23 (0.93)	1 (8)	2 (18)
	Written information on their child's condition	3.00 (1.00)	0	4 (37)
Family presence	Facilitating family presence:			
	During transport	3.67 (0.49)	2 (18)	6 (55)
	On PICU arrival	3.67 (0.65)	6 (55)	2 (18)
	During resuscitation	3.42 (0.79)	3 (27)	3 (27)
Patient needs	Providing patients the ability to request for parents/guardians	3.91 (0.30)	4 (40)	0
	Providing patients the ability to contact parents/guardians	3.91 (0.30)	1 (10)	1 (10)
	Ensuring patient comfort	3.73 (0.47)	5 (50)	1 (10)
	Allowing patient to bring comfort items during transfer	3.09 (0.94)	0	8 (80)
Family needs	Providing the family with:			
	Travel directions and PICU wayfinding	3.50 (0.80)	4 (40)	1 (10)
	Sleeping accommodations	3.42 (0.67)	4 (40)	1 (10)
	PICU information on family resources	3.25 (0.75)	0	0
	Transport assistance	3.17 (0.83)	0	0
	Housing resources	2.83 (0.83)	0	0
	Social work assistance soon after PICU arrival	2.58 (1.00)	1 (10)	1 (10)
	Communication resources	2.58 (1.00)	0	0
	Insurance resources	2.5 (1.00)	1 (10)	2 (20)
	Childcare resources	2.42 (1.00)	0	0
	Spiritual resources	2.17 (1.27)	0	5 (50)

PICU, pediatric intensive care unit.

a Ranked on a Likert scale: 1-not important, 2, somewhat important, 3-important, 4-very important.

Only nine care practices were retained in the second survey per the criteria noted above. Among these, three were rated by majority of respondents (78%) as most important to them: providing the family with verbal information about their child's condition, providing patients the ability to request for parents/guardians throughout the transfer process (if patients are able), and immediately contacting the family if the patient has a significant status change (Table 3).

**Table 3:** Most important patient and family-centered care practices from second survey round.

Domain and care practices^a^	Description	Ranked most important *n* (%)	Ranked least important *n* (%)
		*n* = 9	
Patient/family understanding of need to transfer care			
Ensuring that the family understands:			
Reason for transfer	Care practices to facilitate parents'/guardians' understanding of the reason for their child's transfer to a higher level of care	2 (22)	1 (11)
Emergent nature of transfer	Care practices to facilitate parents'/guardians' understanding of why transfer is emergent and can be potentially life-saving for their child	1 (11)	7 (78)
Communication with patient/family			
*Providing the family with verbal Information on the child's condition* ^b^	*Parents/guardians are verbally informed about their child's condition at both referring and accepting hospitals*	7 (78)	0
*Immediately contacting the family with status change* ^b^	*Parents/guardians are immediately contacted at any time that their child's condition deteriorates*	7 (78)	1 (11)
Providing the family with information on medical procedures	Parents/guardians are given adequate information regarding any emergency procedures performed on their child immediately before or after the procedure at both referring and accepting hospitals	1 (11)	1 (11)
Patient needs			
*Providing patients the ability to request for parents/guardians* ^b^	*The child has the option to request that their parent/guardian be at the bedside at any time at both referring and accepting hospitals*	7 (78)	0
Family needs			
Providing the family with transport assistance	Parents/guardians are given assistance in arranging transportation to the accepting PICU if needed	2 (22)	2 (22)
Providing the family with PICU Information on family resources	Parents/guardians are given information on arrival regarding the PICU's available facilities and resources for families	0	8 (89)
Providing the family with housing resources	Parents/guardians are given resources for housing as needed within the first 24 h of PICU transfer	0	7 (77)

PICU, pediatric intensive care unit.

a Care practices included in the second survey round were ranked as important/very important by ≥75% of participants and were not ranked as unimportant by any participant in the first survey round.

b Care practices ranked overall as most important by majority of parents responding to the second survey round.

### 3.3 Qualitative themes on family experiences of inter-facility transfer

Four parents participated in semi-structured interviews, three of which were conducted via video-conference and one over the phone. All of the parents interviewed traveled separately from their child to the PICU due to logistical/infection control constraints (e.g., lack of space in vehicle, COVID-19 infection control policies) and personal preference. Given the small sample of interviews, we did not achieve thematic saturation.

We identified five main themes describing patients' and families' experiences during the inter-facility transfer process. The most prominent theme was that healthcare teams' communication with families throughout the transfer process played a significant role in families' experiences of inter-facility transfer. Good communication was perceived to lead to meaningful involvement of the family and shared decision-making, while poor communication increased parental stress, anxiety, and frustration. The second theme was family presence. Although parents would like to be with their child at all times during the transfer process and separation was stressful, many understood that this may not be logistically possible for all transfers. Parents, however, expressed the importance of having their child be able to ask for them at any time (if the child is able) during the transfer process, even if parents are unable to immediately be at the bedside. The third theme was related to the unique experiences of families with children who have chronic illness. In many ways, the inter-facility transfer process was familiar to them and they may have had existing relationships with referring and receiving healthcare teams; however, these families experienced additional challenges with regard to transporting medical equipment and ensuring that they were always available to provide information about their child that may not be readily accessible to clinicians. The fourth theme refers to resources and support available to families. Many parents agreed that providing additional support (social work services, housing, transportation, childcare, etc.) was important, though its significance varies depending on the circumstances of the family and prior experiences of PICU transfer and admission. The final theme emphasizes that families' greatest priority above all else was that their child receive adequate medical care and attention even at the expense of up-to-the-moment family communication. These themes and corresponding illustrative quotes are presented in Table 4.

**Table 4:** Qualitative themes on family experiences of inter-facility transfer .

Themes	Representative quotes
Communication with the Healthcare Team	
(1) Good communication between healthcare teams and families can result in meaningful family involvement in decision-making and a seamless care transition.	*“I was heavily involved in the conversations determining whether or not she should be transferred and agreed that that was in my daughter's best interests.”*
	*“And then when (my child) got there, I got to listen to the handoff and all the things that were discussed about the care that she needed and things like that. And I got settled in, met with the doctors, and a big team of nurses, went through everything and. You know, just to make sure everybody is on the same page and everything. So that was very nice.”*
(2) Poor communication regarding the transfer process, the child's clinical condition, and PICU arrival contributes to parental stress and frustration.	*“I was not updated during the transport and never given that option or anything. Very little communication between me and transport hospital about what was going on.”*
	*“One thing… I kind of wish… was that I would get notified when my child got to the university (receiving PICU), because when he got flighted I wasn't able to go with him, and I had no idea if he made it there or if there were any complications…”*
Family Presence	
(3) Parents view separation from their child as a stressful but sometimes necessary part of the transfer process, which they attempt to mitigate in different ways.	*“I mean I think ultimately every parent would love to go with their child if they are going to be life flighted to another hospital, but I just think that is not (always) possible.”*
	*“I think… the helicopter transfer was difficult, but I feel like reassuring him (the patient) when he arrived was better to me. So, he knew I was there, he had someone when he left and somebody when he arrived. I understand why you can't be there in the transfer in the helicopter, I get that.”*
(4) Parents expressed a strong desire to be with their child if their clinical status is unstable and strongly noted the importance of having their child be able to ask for their presence.	*“Yes, it would have been nice to have been able to go with them, because I guess worst case scenario if something were to happen en route, obviously that would have been catastrophic for use not being able to say goodbye.”*
	*“I think it is most important that a parent is provided with the opportunity to be next to their child and informed of their care 24/7. I generally tend to know more about what affects my child (allergies, meds, etc.) and should be in the know of what is happening…as well as be by their side to comfort, love, and support them…”*
Unique experience of families of children with complex chronic conditions^a^	
(5) Families of children who have complex chronic conditions and require frequent inter-facility transfer are more familiar with the process; however, they experience additional challenges.	*“… it helps that I'm very familiar with the (receiving hospital), so I know exactly where to go, where… (to)… check in initially… Unfortunately, we're there a lot, they could even recognize me as well. So I knew where we're going and everything.”*
	*“…we have so much equipment that we need to take with us when we transfer hospitals…”*
	*“…my child's non-verbal. So it's important to me that I hear what's going on… I'm part of that. So I think that goes very well whenever we transfer.”*
Resources and support available to families	
(6) The importance of providing support and resources to families vary depending on the family's circumstances and whether they have prior experience of PICU admission.	*“…that (knowing about available resources) would be very important for patients' families to have because like I said we're not really paying attention to those things, so if someone were to come to use and suggest these things need to be discussed, that would be very beneficial.”*
	*“I would say (knowing about available resources was) not so important, and mainly because we are frequent fliers, so I know not to bring my other child… Okay, I know what services are available. I know our insurance very well. I know all that stuff.”*
Appropriate Care for their Children are Families' Greatest Priority	
(7) Families' greatest priority during the transfer process was that their child was adequately attended to and provided appropriate care by the healthcare team.	*“…when it came down to it, it really wasn't about me—it was about her, and I knew that no matter what I just had to endure it (the transfer process) because this is where she had to be, and I am just thankful that everything worked out okay.”*
	*“The most important thing for us was that our child received the medical attention he needed. Although I wanted to know what was happening I also want the medical team to concentrate on his needs.”*

PICU, pediatric intensive care unit.

a A complex chronic condition is a medical condition that can be reasonably expected to last at least 12 months and to involve either several different organ systems or one organ system severely enough to require specialty pediatric care.

## 4 Discussion

Our study, which aimed to identify care practices important to patients and families during urgent inter-facility transfers of children to a higher level of care, primarily showed that care practices which foster good communication between families and healthcare teams during the transfer are most important to families. Although these findings are exploratory and will need to be confirmed in larger studies, prior work affirms these findings, showing that consistent and empathetic communication between families and referring/receiving clinicians is essential to mitigate families' stress, engage them in shared decision-making, and determine resource needs (4, 10, 24). Moreover, patients/families and multidisciplinary clinicians agree that safe and appropriate care of children during transfers is highly dependent on good communication (25, 26).

Given these findings, future efforts to improve family-centered care during this crucial care transition may need to heavily lean towards ensuring excellent communication with families. One study of family experience during pediatric critical care transports showed that families value clinicians who provide adequate and accurate information while acknowledging parents' anxieties, make families feel involved in their child's care, and convey professionalism and compassion when communicating (27). Fostering good communication in this manner across institutions, through transport, and among various healthcare teams (referring clinicians, receiving PICU team, and transport team) will require partnership and collaboration across our highly siloed health systems. Efforts to improve referring clinician-to-receiving PICU communication and including information about families' preferences and resource needs in inter-facility handoffs may be a worthwhile first step (13). One study has shown success in instituting a “family visit” pre-transport after ED stabilization, which brings together the transport team, ED team, and parents/guardians to update families on patient status, inform them of transport plans, and address concerns regarding the patient's care and transfer process (28). On the other hand, our study also showed that, as much as parents desired communication, they also did not want communication between them and clinicians to delay or interfere in their child's care. Thus, further investigation is needed on how best to integrate family communication during this crucial time (29).

Although facilitating family presence during transport was assessed as one of the least important care practices in the survey, our interviews with parents nevertheless revealed their strong desire to be with their child en route to the PICU, especially if the child was clinically unstable. This apparent contradiction may be explained by sampling differences between surveys and interviews or by parents'/guardians' ability to include more subtlety and complexity in their interview responses vs. closed-ended survey responses; however, a true difference in preference may exist among families, which need to be further explored in future work. In the literature, parental accompaniment during transport has long been considered a key measure of family-centeredness, with many studies showing that family presence results in more positive experiences for the parent and child without hindering necessary medical care or increasing the risk of adverse events (30–32). Despite this, rates of family presence on transport vary widely, which is largely explained by differences in transport policies (33). Although it may not always be feasible for families to accompany children during transport (which parents recognized in our interviews), some authors have suggested that institutions can maximize family presence by defining consistent protocols, establishing a more welcoming attitude towards parents, and implementing education on family-centered care for transport staff (33). During times when families cannot accompany children, standard communication should be implemented so that parents are informed within a reasonable timeframe of significant changes in clinical status and arrival to the PICU.

Our study also highlighted the potential differential impact of inter-facility transfers on patients with complex chronic conditions. Over the past decade, the proportion of children with special healthcare needs has increased to as much as 70% of the PICU population (34, 35). Prehospital care and medical transport have adapted accordingly to address this growing population (36). In a similar vein, the unique needs of these children and their families must be taken into consideration in efforts to improve the family-centeredness of transfers. One parent we interviewed emphasized the added challenge of needing to be available at all times to supply information for their child. Some of this burden may be alleviated by more fully adopting the use of emergency information forms (EIFs), as recommended by the American Academy of Pediatrics and American College of Emergency Physicians. The EIF is a medical summary that describes the child's medical condition, medications, and special care needs to guide clinicians and ensure that the child receives optimal emergency medical care (37).

Although assessment of family needs and the provision of resources is recommended in both PICU and ED guidelines for family-centered care (8, 38), many parents assessed the provision of social work, childcare, or insurance support as relatively less important compared to other care practices. We strongly suggest caution in interpreting this preliminary finding given our small sample which may not be representative of diverse family backgrounds. Respondents themselves noted that additional support may be more important for families with different circumstances. This is especially relevant since inter-facility transfers of hospitalized children disproportionately occur among disadvantaged groups, such as those who have public insurance, belong to lower income quartiles, and/or live in more rural areas (39). Unfortunately, vulnerable populations were underrepresented in our sample, which limited us from assessing their specific needs. Additional research is needed to investigate varying needs across families of different backgrounds and potential disparities in inter-facility transfer care.

Our study has limitations, foremost of which is the limited sample size and attrition across the two surveys and interview due to difficulty in re-contacting patients post-PICU discharge and our inability to provide incentives for survey/interview completion. We were also unable to continue recruitment despite the small sample size due to the study's unfunded nature and limited personnel/resources. This work was also conducted in a single PICU in the Midwest with a mostly rural catchment area. Although we used purposive sampling for broader representation, our sample overrepresented White and non-Hispanic patients/families. Though this is reflective of the area's population (40), we also noted that many underrepresented families did not respond to our contact attempts to administer the surveys. Our findings may thus have been subject to selection and response bias, capturing perspectives of participants who had more time and resources to complete surveys and interviews. We were also unable to include participants who spoke languages other than English due to lack of resources for interpretation. Given all these limitations related to our sample, our findings may not be generalizable to the general PICU population and their experiences across the country. Specifically, our findings may not be generalizable to the experiences of underrepresented populations; we are also unable to delineate differences in the barriers/challenges that these patients and families face and their unique care needs during inter-facility transfer. Most of the survey respondents and interviewees were patients' mothers, therefore we may not have captured the perspectives of other key caregivers such as fathers, siblings, and extended family. Participants who agreed to be interviewed were self-selected and none were able to accompany their child during transport, thus may have been biased in their views of inter-facility transfer.

Despite these limitations, we believe that this exploratory work makes a substantial contribution, given that inadequate attention has been heretofore paid to a crucial transition point for critically ill children and their families. Though preliminary, our findings contribute foundational data for future studies to further delineate families' experiences and to develop structured communication tools, standard workflows, and policy changes ensuring that care during inter-facility transfer is patient- and family-centered. We have also shown that future work to discern disparities in patient/family needs is essential to ensure that specific care practices are targeted towards those who may benefit the most. Overall, these preliminary findings serve as an initial step towards improving inter-facility transfers in ways that matter most to patients and families.

## 5 Conclusions

In this exploratory study, we found that care practices ensuring that timely information about a child's clinical condition is relayed to their parents/guardians were most important to families of critically ill children undergoing inter-facility transfer to the PICU. Communication between families and healthcare team members throughout the transfer process may also play a significant role in families' experiences of transfer. Future directions include expanding this work to include a larger sample of families with more diverse backgrounds in various locations under different pediatric critical care referral networks. Further investigation is also needed to develop interventions to improve communication with families during inter-facility transfers to intensive care.

## Data Availability

The datasets presented in this article are not readily available because we are unable to share data given the high risk of participant identification due to the small sample and inclusion of qualitative responses. Requests to access the datasets should be directed to Christina Cifra, christina.cifra@childrens.harvard.edu.
