# Supplementary material for: Patient and family-centered care practices during inter-facility transfer of critically ill children: an exploratory mixed methods study
Source: Front Health Serv. 2026 Aug 20;6:1835422. doi: 10.3389/frhs.2026.1835422 (PMC13538116; doi:10.3389/frhs.2026.1835422)
Supplement: Supplementary file 1 [file Datasheet1.pdf]

SUPPLEMENTARY MATERIAL

Table of Contents

Supplement to Methods

Supplemental Table 1. Compiled List of Patient and Family-Centered Care Practices Relevant to Inter-facility Transfer of Critically Ill Children to the Pediatric Intensive Care Unit

Supplemental Figure 1. Survey Instrument – Round 1

Supplemental Figure 2. Survey Instrument – Round 2

Supplemental Figure 3. Interview Guide

Supplement to Results

Supplemental Table 2. Patient and Family Characteristics by Survey Response

Supplemental Table 1. Compiled List of Patient and Family-Centered Care Practices Relevant to Inter-facility Transfer of Critically Ill Children to the Pediatric Intensive Care Unit

Domain	Care Practices	Description
Family/patient understanding regarding the patient's condition, reason for transfer, and the transfer process	Ensuring that the family has a good understanding of the:	
	Reason for transfer	Parents/guardians achieve a clear understanding of the reason for transfer to a higher level of care
	Emergent nature of transfer	Patients/guardians understand why transfer is emergent and can be potentially life-saving for the patient
	Risk-benefit of transport	Parents/guardians understand the risks and benefits to transferring hospitals for a higher level of care
	Process of transfer	Parents/guardians achieve a clear understanding of the process of transfer (e.g., communication with accepting hospital/PICU, medical preparations for travel, "loading" into vehicle, transport, arrival to accepting hospital/PICU)
	Providing an opportunity for family to meet the transport crew	Parents/guardians are given the opportunity to meet with transporting medical personnel and ask questions before transfer begins
	Considering the impact of transfer on family	The referring clinician considers the potential impact a transfer may have on the patient and family before making a decision
Communication with family/patient	Providing the family with:	
	Verbal information	Parents/guardians are provided verbal information about their child's condition at the referring and receiving hospital
	Written information	Parents/guardians are provided written information about their child's condition at the referring and receiving hospital
	Updates during transport	Parents/guardians receive relevant updates on their child's status from the transport team en route to the receiving hospital
	PICU contact information	Parents/guardians are provided contact information for the receiving PICU so they can obtain updates on their child while traveling to the hospital
	Information on medical procedures	Parents/guardians are provided adequate information regarding any emergency procedures performed on their child immediately before or after the procedure at the referring and receiving hospital
	Interpreters if needed	Third-party interpreters are present for all discussions between clinicians and parents/guardians who are non-English speaking
	Immediately contacting the family with status change	Parents/guardians are immediately contacted at any time that their child's condition deteriorates
Family presence	Facilitating family presence:	
	During transport	Parents/guardians have the option to accompany their child during transfer if possible
	During resuscitation	If resuscitation efforts are needed at any stage during transfer, parents/guardians have the option to be with/remain with their child
	On PICU arrival	Parents/guardians have the option to be with their child immediately upon arrival to the PICU
Patient needs	Ensuring patient comfort	Patient comfort is a top priority for the entire duration of transfer
	Allowing patient to bring comfort items during transfer	Patients have the option to bring with them comfort items (e.g., stuffed animals, special blankets) from home for the entire duration of the transfer
	Providing patients the ability to request for parents/guardians	Patients have the option to request that their parent/guardian be at the bedside at any time at the referring and receiving hospital
	Providing patients the ability to contact parents/guardians	Patients have the option to contact their parent/guardian at any time at the referring and receiving hospital
Family needs	Providing the family with:	
	Transport assistance	Parents/guardians are provided assistance in arranging transportation to the receiving hospital if needed
	Travel directions and PICU wayfinding	Parents/guardians are provided adequate information for traveling to new hospital and wayfinding to the PICU once at new hospital
	PICU information on family resources	Parents/guardians are provided information on arrival regarding the PICU's available facilities and resources for families
	Social work assistance soon after PICU arrival	A social worker contacts the parents/guardians within 24 hours of PICU admission to provide family resources as applicable
	Housing resources	Parents/guardians are provided resources for housing as needed within the first 24 hours of PICU transfer
	Insurance resources	Parents/guardians are provided financial/insurance resources as needed within the first 24 hours of PICU transfer
	Childcare resources	Parents/guardians are provided resources for child care as needed within the first 24 hours of PICU transfer
	Sleeping accommodations	On arrival to the PICU, the family is provided options for adequate sleeping locations in proximity to the patient
	Spiritual resources	The family has the option to request contact with the hospital chaplain/spirituality team before, during, and after transfer
	Communication resources	Assistance is provided for the family to contact other family members, friends, and other community support

PICU - pediatric intensive care unit

Supplemental Figure 1. Survey Instrument – Round 1

IOWA HEALTH CARE

Introduction

ROUND 1 SURVEY

Thank you for agreeing to participate in this series of surveys to help us improve the care we provide to patients being transferred to the pediatric intensive care unit (PICU).

As someone whose child has been transferred to the PICU for care, we are interested in your personal perspectives on what outcomes are most important to families whose children are sick and needing transfer to a PICU.

If you have any questions or comments, please do not hesitate to contact any of the following research team members:
Claire Stucky: claire-stucky@uiowa.edu
Dr. Christina Cifra: christina-cifra@uiowa.edu
Dr. Aditya Badheka: aditya-badheka@uiowa.edu

In this survey, you will be presented with specific outcomes or events that may occur surrounding transfer of a critically ill child from a referring hospital to another hospital for admission to the PICU.

Please feel free to refer to your past experience/s with your child's transfer/s to the PICU in order to indicate which outcomes you think are most important to children and their families during the period of time surrounding interhospital transfer.

A. Patient/Family Understanding Regarding Need to Transfer Care

PATIENT / FAMILY UNDERSTANDING REGARDING NEED TO TRANSFER CARE

Please indicate how important you think each outcome is to patients and their families.

	Not important	Somewhat Important	Important	Very Important
**Understanding Risk-Benefit of Transport**: Parents/guardians understand the risks and benefits of transporting their child to a higher level of care.	○	○	○	○

	Not important	Somewhat Important	Important	Very Important
**Understanding Emergent Nature of Transfer**: Parents/guardians understand why transfer is emergent and can be potentially life-saving for their child.	○	○	○	○

	Not important	Somewhat Important	Important	Very Important
**Understanding Process of Transfer**: Parents/guardians understand the process of transferring their child (e.g., communication with accepting PICU, medical preparations for travel, "loading" into vehicle, transport, arrival to accepting PICU).	○	○	○	○

	Not important	Somewhat Important	Important	Very Important
**Understanding that Family Impact was Considered**: It is clear to parents/guardians that referring clinicians have considered the potential impact of transfer to the patient's family.	○	○	○	○

	Not important	Somewhat Important	Important	Very Important
**Understanding Reason for Transfer**: Parents/guardians understand the reason for their child's transfer to a higher level of care.	○	○	○	○

	Not important	Somewhat Important	Important	Very Important
**Meeting Transport Crew**: Parents/guardians have the opportunity to meet with transporting medical personnel and ask questions before transport.	○	○	○	○

Which outcome do you think is overall **MOST important** to patients and their families?

- ○ **Understanding Process of Transfer:** Parents/guardians understand the process of transferring their child (e.g., communication with accepting PICU, medical preparations for travel, "loading" into vehicle, transport, arrival to accepting PICU).
- ○ **Understanding that Family Impact was Considered:** It is clear to parents/guardians that referring clinicians understand the potential impact of transfer to their family and have considered this in their decision-making to transfer the patient.
- ○ **Meeting Transport Crew:** Parents/guardians have the opportunity to meet with transporting medical personnel and ask questions before transport.
- ○ **Understanding Emergent Nature of Transfer:** Parents/guardians understand why transfer is emergent and can be potentially life-saving for their child.
- ○ **Understanding Risk-Benefit of Transport:** Parents/guardians understand the risks and benefits of transporting their child to a higher level of care.
- ○ **Understanding Reason for Transfer:** Parents/guardians understand the reason for their child's transfer to a higher level of care.

Which outcome do you think is overall **LEAST important** to patients and their families?

- ○ **Understanding Risk-Benefit of Transport:** Parents/guardians understand the risks and benefits of transporting their child to a higher level of care.
- ○ **Understanding that Family Impact was Considered:** It is clear to parents/guardians that referring clinicians understand the potential impact of transfer to their family and have considered this in their decision-making to transfer the patient.
- ○ **Understanding Reason for Transfer:** Parents/guardians understand the reason for their child's transfer to a higher level of care.
- ○ **Understanding Process of Transfer:** Parents/guardians understand the process of transferring their child (e.g., communication with accepting PICU, medical preparations for travel, "loading" into vehicle, transport, arrival to accepting PICU).
- ○ **Meeting Transport Crew:** Parents/guardians have the opportunity to meet with transporting medical personnel and ask questions before transport.
- ○ **Understanding Emergent Nature of Transfer:** Parents/guardians understand why transfer is emergent and can be potentially life-saving for their child.

Do you have any **comments about any of the outcomes**

included in this section?

Are there any **additional important outcomes** which were not included in this section? If so, please indicate these in the text box provided.

B. Communication with Patient/Family

COMMUNICATION WITH PATIENT / FAMILY

Please indicate how important you think each outcome is to patients and their families.

	Not important	Somewhat Important	Important	Very Important
**Updated During Transport:** Parents/guardians receive at least one update on their child's status from the medical transport team en route to the accepting hospital.	○	○	○	○
**Immediate Contact with Status Change:** Parents/guardians are immediately contacted at any time that their child's condition deteriorates.	○	○	○	○
**Verbal Information Provided:** Parents/guardians are verbally informed about their child's condition at both referring and accepting hospitals.	○	○	○	○

	Not important	Somewhat Important	Important	Very Important
**PICU Contact Information Provided:** Parents/guardians are given contact information for the accepting PICU so they can obtain updates on their child while traveling to the hospital.	○	○	○	○

	Not important	Somewhat Important	Important	Very Important
**Translators Provided:** Translators are present for all discussions between clinicians and parents/guardians who are non-English speaking.	○	○	○	○

	Not important	Somewhat Important	Important	Very Important
**Information on Procedures Provided:** Parents/guardians are given adequate information regarding any emergency procedures performed on their child immediately before or after the procedure at both referring and accepting hospitals.	○	○	○	○

	Not important	Somewhat Important	Important	Very Important
**Written Information Provided:** Parents/guardians are given written information about their child's condition at both referring and accepting hospitals.	○	○	○	○

Which outcome do you think is overall **MOST important** to patients and their families?

- ○ **Verbal Information Provided:** Parents/guardians are verbally informed about their child's condition at both referring and accepting hospitals.
- ○ **Updated During Transport:** Parents/guardians receive at least one update on their child's status from the medical transport team en route to the accepting hospital.
- ○ **Immediate Contact with Status Change:** Parents/guardians are immediately contacted at any time that their child's condition deteriorates.
- ○ **Information on Procedures Provided:** Parents/guardians are given adequate information regarding any emergency procedures performed on their child immediately before or after the procedure at both referring and accepting hospitals.
- ○ **Written Information Provided:** Parents/guardians are given written information about their child's condition at both referring and accepting hospitals.

- ○ **PICU Contact Information Provided:** Parents/guardians are given contact information for the accepting PICU so they can obtain updates on their child while traveling to the hospital.
- ○ **Translators Provided:** Translators are present for all discussions between clinicians and parents/guardians who are non-English speaking.

Which outcome do you think is overall **LEAST important** to patients and their families?

- ○ **Immediate Contact with Status Change:** Parents/guardians are immediately contacted at any time that their child's condition deteriorates.
- ○ **Verbal Information Provided:** Parents/guardians are verbally informed about their child's condition at both referring and accepting hospitals.
- ○ **Information on Procedures Provided:** Parents/guardians are given adequate information regarding any emergency procedures performed on their child immediately before or after the procedure at both referring and accepting hospitals.
- ○ **PICU Contact Information Provided:** Parents/guardians are given contact information for the accepting PICU so they can obtain updates on their child while traveling to the hospital.
- ○ **Translators Provided:** Translators are present for all discussions between clinicians and parents/guardians who are non-English speaking.
- ○ **Written Information Provided:** Parents/guardians are given written information about their child's condition at both referring and accepting hospitals.
- ○ **Updated During Transport:** Parents/guardians receive at least one update on their child's status from the medical transport team en route to the accepting hospital.

Do you have any **comments about any of the outcomes** included in this section?

Are there any **additional important outcomes** which were not included in this section? If so, please indicate these in the text box provided.

C. Family Presence

FAMILY PRESENCE

Please indicate how important you think each outcome is to patients and their families.

	Not important	Somewhat Important	Important	Very Important
**Presence During Resuscitation** (outside of transport)**:** If rescuscitation efforts such as CPR are needed at any stage during transfer, parents/guardians have the option to be with/remain with their child.	○	○	○	○
**Presence During Transport:** Parents/guardians have the option to accompany their child during transport if possible.	○	○	○	○
**Presence on PICU Arrival:** Parents/guardians have the option to be with their child immediately upon their arrival to the PICU.	○	○	○	○

Which outcome do you think is overall **MOST important** to patients and their families?

- ○ **Presence During Transport:** Parents/guardians have the option to accompany their child during transport if possible.
- ○ **Presence During Resuscitation:** If rescuscitation efforts are needed at any stage during transfer, parents/guardians have the option to be with/remain with their child.
- ○ **Presence on PICU Arrival:** Parents/guardians have the option to be with their child immediately upon their arrival to the PICU.

Which outcome do you think is overall **LEAST important** to patients and their families?

- ○ **Presence on PICU Arrival:** Parents/guardians have the option to be with their child immediately upon their arrival to the PICU.
- ○ **Presence During Resuscitation:** If rescuscitation efforts are needed at any stage during transfer, parents/guardians have the option to be with/remain with their child.
- ○ **Presence During Transport:** Parents/guardians have the option to accompany their child during transport if possible.

Do you have any **comments about any of the outcomes** included in this section?

Are there any **additional important outcomes** which were not included in this section? If so, please indicate these in the text box provided.

D. Patient Needs

PATIENT NEEDS

Please indicate how important you think each outcome is to patients and their families.

	Not important	Somewhat Important	Important	Very Important
**Ability to Request for Parents/Guardians:** The child has the option to request that their parent/guardian be at the bedside at any time at both referring and accepting hospitals.	○	○	○	○
**Patient Comfort:** The child's comfort is a top priority for the entire duration of transfer.	○	○	○	○
**Child Can Contact Parents/Guardians:** The child has the option to contact their parent/guardian at any time at both referring and accepting hospitals.	○	○	○	○
**Bringing Comfort Items:** The child has the option to bring with them comfort items (e.g., stuffed animals, special blankets) from home for the entire duration of the transfer.	○	○	○	○

Which outcome do you think is overall **MOST important** to patients and their families?

- ○ **Patient Comfort:** The child's comfort is a top priority for the entire duration of transfer.
- ○ **Child Can Contact Parents/Guardians:** The child has the option to contact their parent/guardian at any time at both referring and accepting hospitals.
- ○ **Ability to Request for Parents/Guardians:** The child has the option to request that their parent/guardian be at the bedside at any time at both referring and accepting hospitals.
- ○ **Bringing Comfort Items:** The child has the option to bring with them comfort items (e.g., stuffed animals, special blankets) from home for the entire duration of the transfer.

Which outcome do you think is overall **LEAST important** to patients and their families?

- ○ **Child Can Contact Parents/Guardians:** The child has the option to contact their parent/guardian at any time at both referring and accepting hospitals.
- ○ **Ability to Request for Parents/Guardians:** The child has the option to request that their parent/guardian be at the bedside at any time at both referring and accepting hospitals.
- ○ **Patient Comfort:** The child's comfort is a top priority for the entire duration of transfer.
- ○ **Bringing Comfort Items:** The child has the option to bring with them comfort items (e.g., stuffed animals, special blankets) from home for the entire duration of the transfer.

Do you have any **comments about any of the outcomes** included in this section?

Are there any **additional important outcomes** which were not included in this section? If so, please indicate these in the text box provided.

E. Family Needs

FAMILY NEEDS

Please indicate how important you think each outcome is to patients and their families.

	Not important	Somewhat Important	Important	Very Important
**Transport Assistance Provided:** Parents/guardians are given assistance in arranging transportation to the accepting PICU if needed.	○	○	○	○
**Travel Directions and PICU Wayfinding Assistance Provided:** Parents/guardians are given information for traveling to the accepting hospital and PICU wayfinding.	○	○	○	○
**PICU Information on Family Resources Provided:** Parents/guardians are given information on arrival regarding the PICU's available facilities and resources for families.	○	○	○	○

	Not important	Somewhat Important	Important	Very Important
**Social Work Contact:** A social worker contacts the parents/guardians within 24 hours of PICU admission to provide family resources as applicable.	○	○	○	○
**Housing Resources Provided:** Parents/gaurdians are given resources for housing as needed within the first 24 hours of PICU transfer.	○	○	○	○
**Insurance Resources Provided:** Parents/guardians are given financial/insurance resources as needed within the first 24 hours of PICU transfer.	○	○	○	○

	Not important	Somewhat Important	Important	Very Important
**Child Care Resources Provided:** Parents/guardians are given resources for child care as needed within the first 24 hours of PICU transfer.	○	○	○	○

	Not important	Somewhat Important	Important	Very Important
**Sleeping Accommodations Provided:** On arrival to the PICU, parents/guardians are given options for adequate sleeping locations in proximity to the patient.	○	○	○	○

	Not important	Somewhat Important	Important	Very Important
**Spiritual Resources Provided:** The family has the option to request contact with the hospital chaplain/spirituality team before, during, and after transfer.	○	○	○	○

	Not important	Somewhat Important	Important	Very Important
**Communication Resources Provided:** Assistance is given for the family to contact other family members, friends, and other community support.	○	○	○	○

Which outcome do you think is overall **MOST important** to patients and their families?

- ○ **Sleeping Accommodations Provided:** On arrival to the PICU, parents/guardians are given options for adequate sleeping locations in proximity to the patient.
- ○ **Transport Assistance Provided:** Parents/guardians are given assistance in arranging transportation to the accepting PICU if needed.
- ○ **Spiritual Resources Provided:** The family has the option to request contact with the hospital chaplain/spirituality team before, during, and after transfer.
- ○ **Child Care Resources Provided:** Parents/guardians are given resources for child care as needed within the first 24 hours of PICU transfer.
- ○ **Communication Resources Provided:** Assistance is given for the family to contact other family members, friends, and other community support.
- ○ **Insurance Resources Provided:** Parents/guardians are given financial/insurance resources as needed within the first 24 hours of PICU transfer.
- ○ **Travel Directions and PICU Wayfinding Assistance Provided:** Parents/guardians are given information for traveling to the accepting hospital and PICU wayfinding.

- ○ **Social Work Contact:** A social worker contacts the parents/guardians within 24 hours of PICU admission to provide family resources as applicable.
- ○ **Housing Resources Provided:** Parents/gaurdians are given resources for housing as needed within the first 24 hours of PICU transfer.
- ○ **PICU Information on Family Resources Provided:** Parents/guardians are given information on arrival regarding the PICU's available facilities and resources for families.

Which outcome do you think is overall **LEAST important** to patients and their families?

- ○ **Insurance Resources Provided:** Parents/guardians are given financial/insurance resources as needed within the first 24 hours of PICU transfer.
- ○ **Transport Assistance Provided:** Parents/guardians are given assistance in arranging transportation to the accepting PICU if needed.
- ○ **Child Care Resources Provided:** Parents/guardians are given resources for child care as needed within the first 24 hours of PICU transfer.
- ○ **PICU Information on Family Resources Provided:** Parents/guardians are given information on arrival regarding the PICU's available facilities and resources for families.
- ○ **Spiritual Resources Provided:** The family has the option to request contact with the hospital chaplain/spirituality team before, during, and after transfer.
- ○ **Sleeping Accommodations Provided:** On arrival to the PICU, parents/guardians are given options for adequate sleeping locations in proximity to the patient.
- ○ **Housing Resources Provided:** Parents/gaurdians are given resources for housing as needed within the first 24 hours of PICU transfer.
- ○ **Social Work Contact:** A social worker contacts the parents/guardians within 24 hours of PICU admission to provide family resources as applicable.
- ○ **Travel Directions and PICU Wayfinding Assistance Provided:** Parents/guardians are given information for traveling to the accepting hospital and PICU wayfinding.
- ○ **Communication Resources Provided:** Assistance is given for the family to contact other family members, friends, and other community support.

Do you have any **comments about any of the outcomes** included in this section?

[ ]

Are there any **additional important outcomes** which were not included in this section? If so, please indicate these in the text box provided.

[ ]

Supplemental Figure 2. Survey Instrument – Round 2

IOWA
HEALTH CARE

Introduction

ROUND 2 SURVEY

Thank you again for agreeing to participate in this series of surveys to help us improve the care we provide to patients being transferred to the pediatric intensive care unit (PICU). We again ask that you please complete this shorter ROUND 2 survey, which has been consolidated to reflect the results of the first survey.

As before, we are interested in your personal perspectives on what outcomes are most important to families whose children are sick and needing transfer to a PICU.

If you have any questions or comments, please do not hesitate to contact any of the following research team members:
Claire Stucky: claire-stucky@uiowa.edu
Dr. Christina Cifra: christina-cifra@uiowa.edu
Dr. Aditya Badheka: aditya-badheka@uiowa.edu

In this survey, you will be presented with specific outcomes or events that may occur surrounding transfer of a critically ill child from a referring hospital to another hospital for admission to the PICU.

Please feel free to refer to your past experience/s with your child's transfer/s to the PICU in order to indicate which outcomes you think are most important to children and their families during the period of time surrounding interhospital transfer.

Block 1

Please indicate how important you think each outcome is to patients and their families.

	Not important	Somewhat Important	Important	Very Important
**Understanding Reason for Transfer**: Parents/guardians understand the reason for their child's transfer to a higher level of care.	○	○	○	○
**Verbal Information Provided:** Parents/guardians are verbally informed about their child's condition at both referring and accepting hospitals.	○	○	○	○
**PICU Information on Family Resources Provided:** Parents/guardians are given information on arrival regarding the PICU's available facilities and resources for families.	○	○	○	○

Which outcome do you think is overall **MOST important** to patients and their families?

- ○ **PICU Information on Family Resources Provided:** Parents/guardians are given information on arrival regarding the PICU's available facilities and resources for families.
- ○ **Understanding Reason for Transfer:** Parents/guardians understand the reason for their child's transfer to a higher level of care.
- ○ **Verbal Information Provided:** Parents/guardians are verbally informed about their child's condition at both referring and accepting hospitals.

Which outcome do you think is overall **LEAST important** to patients and their families?

- ○ **PICU Information on Family Resources Provided:** Parents/guardians are given information on arrival regarding the PICU's available facilities and resources for families.
- ○ **Verbal Information Provided:** Parents/guardians are verbally informed about their child's condition at both referring and accepting hospitals.
- ○ **Understanding Reason for Transfer:** Parents/guardians understand the reason for their child's transfer to a higher level of care.

Do you have any **comments about any of the outcomes** included in this section?

Block 2

Please indicate how important you think each outcome is to patients and their families.

	Not important	Somewhat Important	Important	Very Important
**Information on Procedures Provided:** Parents/guardians are given adequate information regarding any emergency procedures performed on their child immediately before or after the procedure at both referring and accepting hospitals.	○	○	○	○
**Immediate Contact with Status Change:** Parents/guardians are immediately contacted at any time that their child's condition deteriorates.	○	○	○	○
**Understanding Emergent Nature of Transfer**: Parents/guardians understand why transfer is emergent and can be potentially life-saving for their child.	○	○	○	○

Which outcome do you think is overall **MOST important** to patients and their families?

- ○ **Information on Procedures Provided:** Parents/guardians are given adequate information regarding any emergency procedures performed on their child immediately before or after the procedure at both referring and accepting hospitals.
- ○ **Understanding Emergent Nature of Transfer**: Parents/guardians understand why transfer is emergent and can be potentially life-saving for their child.
- ○ **Immediate Contact with Status Change:** Parents/guardians are immediately contacted at any time that their child's condition deteriorates.

Which outcome do you think is overall **LEAST important** to patients and their families?

- ○ **Understanding Emergent Nature of Transfer**: Parents/guardians understand why transfer is emergent and can be potentially life-saving for their child.
- ○ **Immediate Contact with Status Change:** Parents/guardians are immediately contacted at any time that their child's condition deteriorates.
- ○ **Information on Procedures Provided:** Parents/guardians are given adequate information regarding any emergency procedures performed on their child immediately before or after the procedure at both referring and accepting hospitals.

Do you have any **comments about any of the outcomes** included in this section?

Block 3

Please indicate how important you think each outcome is to patients and their families.

	Not important	Somewhat Important	Important	Very Important
**Transport Assistance Provided:** Parents/guardians are given assistance in arranging transportation to the accepting PICU if needed.	○	○	○	○
**Housing Resources Provided:** Parents/gaurdians are given resources for housing as needed within the first 24 hours of PICU transfer.	○	○	○	○
**Ability to Request for Parents/Guardians:** The child has the option to request that their parent/guardian be at the bedside at any time at both referring and accepting hospitals.	○	○	○	○

Which outcome do you think is overall **MOST important** to patients and their families?

- ○ **Ability to Request for Parents/Guardians:** The child has the option to request that their parent/guardian be at the bedside at any time at both referring and accepting hospitals.
- ○ **Housing Resources Provided:** Parents/gaurdians are given resources for housing as needed within the first 24 hours of PICU transfer.
- ○ **Transport Assistance Provided:** Parents/guardians are given assistance in arranging transportation to the accepting PICU if needed.

Which outcome do you think is overall **LEAST important** to patients and their families?

- ○ **Housing Resources Provided:** Parents/gaurdians are given resources for housing as needed within the first 24 hours of PICU transfer.
- ○ **Transport Assistance Provided:** Parents/guardians are given assistance in arranging transportation to the accepting PICU if needed.
- ○ **Ability to Request for Parents/Guardians:** The child has the option to request that their parent/guardian be at the bedside at any time at both referring and accepting hospitals.

Do you have any **comments about any of the outcomes** included in this section?

Final Comments

Please note any other comments you may have regarding this survey.

Supplemental Figure 3. Interview Guide

INTERVIEW GUIDE

Thank you very much for interviewing with us today. We greatly appreciate your time.

As a reminder, the purpose of this interview is to gain more insight into what is important for patients and their families when they require transfer to the pediatric intensive care unit from another facility. We are specifically interested in your experience of the transfer process for your child and how you think we can improve the process for future patients.

Please note that the interview will be audio-recorded so we can create a written transcript for data analysis. When we transcribe this interview and report our results, your name and your child's name will remain confidential and will not be disclosed to anyone. You can also choose not to answer any question. Is it okay to proceed? *(If yes)* I will start the recording now.

1. Can you describe your transfer experience to the pediatric intensive care unit (PICU) at the University of Iowa? This includes the time period from when you were told your child needs to be transferred up to the time you arrived at the PICU. If your child has been urgently transferred to the PICU multiple times, can you describe your most recent experience?

2. What went well with your experience of being transferred to the PICU? What could have been improved? Why?

3. Were there things you wish were available to you and your child during your transfer experience? If so, what were these?

4. From your perspective as a parent/guardian, what do you think is the most important thing that the healthcare team (both at the referring hospital and at the University of Iowa) need to pay attention to for patients being urgently transferred across institutions? Why so?

Through a survey, we asked many families who have had the experience of a child needing urgent transfer to the PICU what was important to them during the time of transfer.

5. Many parents/guardians indicated that the following were NOT important: being present with their child during transfer, being present with their child during any resuscitation effort throughout the transfer process, and being present with their child upon arrival to the PICU. Is being present with your child during any of the mentioned circumstances very important or not so important to you? Why or why not?

6. Parents/guardians were split as to the importance of the PICU providing support to families at the time of their child's admission. This support is in the form of social work services, childcare resources, housing/transportation assistance, and assistance in navigating insurance issues. Are these support services very important or not so important to you? Why or why not?

7. Most parents/guardians indicated that the healthcare team providing them information about their child's condition is very important, even if they do not fully understand the reason for urgent transfer. Would you agree with this? Why or why not?

8. Most parents/guardians indicated that their child having the ability to request for their parents/guardians at any time during the transfer process is very important. Would you agree with this? Why or why not?

9. Would you like to let us know anything else about your transfer experience? Do you have any questions?

That concludes our interview. We are looking forward to improving the transfer experience for our patients and their families, thanks to your participation and feedback.

Thank you very much! I will stop the recording now.

Supplemental Table 2. Patient and Family Characteristics by Survey Response

Characteristics	Responded n=13	Not Responded n=10
**Patient Characteristics**		
Age, years, median (IQR)	4 (0.7, 10)	2 (1, 7)
Female gender, n (%)	4 (31)	3 (30)
Race, n (%)		
White	9 (69)	5 (50)
Black	0	3 (30)
Asian	1 (8)	0
More than one race	3 (23)	0
Ethnicity is Hispanic or Latino, n (%)	2 (15)	0
Has a complex chronic condition, n (%)	8 (62)	5 (50)
Had at least one PICU admission in previous 2 years, n (%)	3 (23)	2 (20)
**Parent/Guardian Characteristics**		
Female gender, n (%)	10 (77)	8 (80)
Relationship to patient, n (%)		
Mother	10 (77)	6 (60)
Father	3 (23)	2 (20)
Grandparent	0	2 (20)
Highest educational attainment, n (%)		
High school	3 (23)	6 (60)
College	8 (62)	3 (30)
Post-graduate	2 (15)	1 (10)
Employment status, n (%)		
Part-time	3 (23)	2 (20)
Full time	9 (69)	4 (40)
Unemployed	1 (8)	4 (40)
**Family Characteristics**		
Median household income by zip code, n (%)		
$35,000 to $49,999	1 (8)	2 (20)
$50,000 to $74,999	8 (62)	7 (70)
$75,000 to $99,999	4 (31)	1 (10)
Geographic distance from PICU, miles, median (IQR)	71 (54, 88)	76.5 (41, 81)
Ground travel time from patient's home to PICU, mins, median (IQR)	80 (60, 90)	82.5 (60, 90)

IQR - interquartile range, PICU - pediatric intensive care unit
